# Handwashing, sanitation and family planning practices are the strongest underlying determinants of child stunting in rural indigenous communities of Jharkhand and Odisha, Eastern India: a cross‐sectional study

**DOI:** 10.1111/mcn.12323

**Published:** 2016-06-27

**Authors:** Jennifer Saxton, Shibanand Rath, Nirmala Nair, Rajkumar Gope, Rajendra Mahapatra, Prasanta Tripathy, Audrey Prost

**Affiliations:** ^1^ UCL Institute for Global Health London UK; ^2^ Ekjut Chakradharpur Jharkhand India

**Keywords:** child stunting, indigenous communities, Eastern India

## Abstract

The World Health Organisation has called for global action to reduce child stunting by 40% by 2025. One third of the world's stunted children live in India, and children belonging to rural indigenous communities are the worst affected. We sought to identify the strongest determinants of stunting among indigenous children in rural Jharkhand and Odisha, India, to highlight key areas for intervention.

We analysed data from 1227 children aged 6–23.99 months and their mothers, collected in 2010 from 18 clusters of villages with a high proportion of people from indigenous groups in three districts. We measured height and weight of mothers and children, and captured data on various basic, underlying and immediate determinants of undernutrition. We used Generalised Estimating Equations to identify individual determinants associated with children's height‐for‐age *z*‐score (HAZ; *p* < 0.10); we included these in a multivariable model to identify the strongest HAZ determinants using backwards stepwise methods.

In the adjusted model, the strongest protective factors for linear growth included cooking outdoors rather than indoors (HAZ +0.66), birth spacing ≥24 months (HAZ +0.40), and handwashing with a cleansing agent (HAZ +0.32). The strongest risk factors were later birth order (HAZ −0.38) and repeated diarrhoeal infection (HAZ −0.23).

Our results suggest multiple risk factors for linear growth faltering in indigenous communities in Jharkhand and Odisha. Interventions that could improve children's growth include reducing exposure to indoor air pollution, increasing access to family planning, reducing diarrhoeal infections, improving handwashing practices, increasing access to income and strengthening health and sanitation infrastructure.

## Introduction

The World Health Organisation has called for global action to reduce child stunting by 40% by 2025 (de Onis et al. 2013). The Lancet's 2013 Maternal and Child Undernutrition Series identified 10 nutrition‐specific interventions that could reduce stunting by 20% if they reached 90% of children in 34 countries (Bhutta et al. 2013). These included strengthening infant and young child feeding practices, providing periconceptional folic acid, energy protein, multiple micronutrients and calcium supplementation to mothers, Vitamin A to children and scaling up the management of moderate and severe acute malnutrition. Improving adolescent nutrition and access to family planning would further contribute to stunting reduction, as would handwashing promotion, and access to safe water and sanitation (Sachdev et al. 2013, Humphrey, 2009, Sachdev, 2012, Spears et al. 2013, Dangour et al. 2013). Nutrition‐sensitive interventions to support women's empowerment, agriculture, food systems, education, employment, social protection and safety nets would provide additional leverage against the underlying and basic causes of undernutrition (Ruel and Alderman, 2013). Scaling up nutrition‐specific and sensitive interventions will improve children's development, schooling attainment and later adult health (Adair et al. 2013).

Approximately one third of the 165 million children affected by stunting in low and middle‐income countries live in India (Black et al. 2013b). A recent analysis of nationally representative data confirmed that influences on growth in this context are multiple, including socio‐economic characteristics and factors related to the environment, nutrition, infections and access to healthcare (Fenske et al. 2013). Further analysis of data from over 26 000 children in the nationally representative NFHS‐3 by Corsi et al. (2015) found that maternal stature, education, household wealth, dietary diversity and maternal BMI were the five most important risk factors for stunting, and accounted for 67.2% of the Population Attributable Risk for stunting. Children from the poorest families and from Scheduled Caste and Scheduled Tribe (indigenous) communities are the worst affected by stunting (IIPS and Macro International, 2007, HUNGaMA Survey Report, 2011). In India's 2013–2014 nationally representative rapid survey of children, 42.3% of children under‐five from Scheduled Tribes were stunted, rising to 64.1% and 53.4% in Jharkhand and Odisha, two states with a high proportion of Scheduled Tribe households (Ministry of Women and Child Development, Government Of India, 2015). Unfortunately, most studies on undernutrition among Scheduled Tribe communities use national data from 2005/6 or small sample sizes, and often do not investigate stunting determinants systematically (Arnold et al. 2009, Debnath and Bhattacharjee, 2014, UNICEF, 2014). Determinants research could help prioritise interventions for scaling up stunting reduction in areas with indigenous populations.

We conducted an analysis of determinants of stunting in over one thousand children in three rural districts of Jharkhand and Odisha with a large proportion of indigenous people, to understand what actions could accelerate stunting reduction in those areas.

**Key messages**

- We identified potentially modifiable stunting determinants to inform a context‐specific stunting reduction strategy for indigenous areas of eastern India.
- There is a clear role for interventions to promote handwashing and reduce diarrhoea. Community‐wide sanitation programmes to promote safe faeces disposal could be valuable, in conjunction with investments in health and sanitation infrastructure.
- Cooking outdoors was positively associated with height‐for‐age. Reducing indoor air pollution from burning biomass fuels could reduce stunting.
- Adequate birth spacing and interventions to minimise unintended pregnancy could reduce stunting.
- Income‐generation and social protection schemes could help households increase dietary diversity.

## Participants and methods

### Study area and participants

We conducted a cross‐sectional nutrition survey in 18 geographic clusters within three districts of Jharkhand and Odisha, two states of eastern India. Both states are largely rural, and many of the village clusters in this survey were in remote, hilly, forested areas. The largest indigenous groups in the study districts are the Ho, Santhal, Munda and Oraon.

Participants were children aged eight weeks to three years and their mothers. We first conducted a census to identify all children under three years of age and women who were more than six months pregnant (who would have delivered by the time we conducted the nutrition survey) to generate the sampling list. If women had more than one eligible child, both siblings were eligible to take part. We excluded multiple births, and children whose mothers had died.

### Data collection

Locally recruited growth monitors and helpers collected data between January and June 2010. Each growth monitor and their helper worked as a pair in the cluster they resided in. All growth monitors took part in a six‐day residential training course, including an anthropometry standardisation exercise recommended for SMART emergency nutrition surveys (ENA for SMART, 2007).

We interviewed mothers in Hindi, Ho or Oriya using a questionnaire capturing the following information: maternal and household socio‐demographic characteristics; household environment, standard of living, water source, sanitation; maternal dietary adequacy and diversity; pregnancy history, antenatal, perinatal and postnatal information; maternal physical and mental health; current childhood illness symptoms (diarrhoea/fever/cough); healthcare seeking for childhood illnesses; breastfeeding and complementary feeding; and contact with health workers. Maternal psychological distress (none/mild, moderate and severe) was measured with the Kessler‐10 (Kessler et al. 2002). The K10 is a validated screening tool and was used locally in a previous study (Tripathy et al. 2010). Maternal and child anthropometry was measured using: SECA 874 weighing scales with taring button (graduation weight: 50 g < 150 kg > 100 g), Leicester height measures (nearest 1 mm), SECA measuring mat for children under two years or unable to stand (nearest 5 mm); Mid‐to‐Upper‐Arm‐Circumference (MUAC) was measured using UNICEF colour‐banded tapes (nearest 1 mm).

### Sample size

The survey from which our data are drawn was part of an evaluation of an intervention to improve maternal and child health and nutrition through participatory women's group meetings. The evaluation involved 36 clusters, 18 of which were control areas. The sample size was calculated to enable detection of 0.2 difference in weight‐for‐height *z*‐scores between the intervention and control clusters, and also took into account potential clustering using two published intraclass correlation coefficients (ICC) for weight‐for‐height from similar studies: 0.017 and 0.054 (Patel et al. 2003, Rahman et al. 2008). We used an intermediary ICC of 0.032–0.034, at the 5% significance level, 80% power and a standard deviation of 1 for both intervention and control groups. We increased the planned sample size by 20% to account for attrition because of seasonal migration. The overall required sample size was *n* = 5184, so we randomly sampled 144 children from the sampling lists in each cluster. For the analysis reported here, we only used data from the 18 control clusters in order not to bias estimates through any potential effects of the intervention. We only included children aged 6–23.99 months, which is a commonly used age group for analysing linear growth determinants, enabling comparison of our results with other studies.

### Data management and analysis

We collected data on paper forms and entered them twice into an Access database. Discrepancies between the first and second entries were resolved by returning to the original record. We examined anthropometric data for completeness and plausibility using ENA for SMART software before generating *z*‐scores, then used SPSS version 21 for all subsequent analyses.

#### Statistical analysis

The analysis of potential stunting determinants was carried out in three steps. First, we identified potential determinants of child undernutrition using the UNICEF conceptual framework to test in the models (UNICEF, 1990). The framework maps determinants hierarchically as distal basic causes (e.g. poverty, governance), underlying causes (e.g. food security, child caring practices, health services and environment) and immediate causes (breastfeeding , disease). The questionnaire was designed to mainly capture information on determinants at the immediate and underlying level, and less on determinants at the basic level. Table 1 shows all potential determinants for which data were collected. We used Generalised Estimation Equation (GEE) analysis to assess the univariable association between each potential determinant with HAZ, retaining those with significance values of *p* < 0.1 (see Additional File 1).

**Table 1:** Potential determinants of undernutrition and corresponding study variables, classified according to the UNICEF conceptual framework

Determinant category	Variables
**Basic causes**	Socioeconomic quintile^a^
	Income group^b^
	Maternal education
	Father's education
	Social group (Scheduled Tribe, Scheduled Caste, Other Backward Class)
	Religion
	District
	Relationship to household head
**Underlying causes**	
Household shocks	Household shocks in the last 12 months^c^
Maternal health	Parity/Birth spacing
	Self‐reported anaemia and malaria in pregnancy
	Self‐reported food intake during pregnancy
	Iron tablet consumption during pregnancy
	Maternal BMI/Maternal height
	Non‐pregnancy related illness/injury in the last three months
	Psychological distress (last four weeks)
Child health and feeding practices	Early initiation of breastfeeding
	Pre‐lacteal feeds
	Bottle‐feeding
	Colostrum discarding/BCG, DPT and Polio immunisations
	Feeding and treatment seeking during childhood illness
	Use of oral rehydration solution for child diarrhoea
	Birth order
Underlying child health issues	Repeated attacks of diarrhoea, fever and cough
Health environment and services	Place of delivery
	Antenatal and postnatal visits
	Growth monitoring and food ration provision through the Anganwadi Centre
	Sufficient living area (≤3 people per sleeping room)
	Cooking location (main living area, separate room or outdoors)
	Season of birth
	Treatment of drinking water
	Source of drinking water
	Accessibility of drinking water (≤30 min)
	Disposal of children's faeces
	Use of a handwashing agent (soap/ash/mud)
	Occasions when cleansing agent is used for handwashing (before preparing food/feeding a child/eating, after defecation/cleaning up a child who has defecated)
**Immediate causes**
Dietary intake/breastfeeding (previous 24 h)	Predominant breastfeeding
	Age‐appropriate breastfeeding
	Minimum dietary diversity (≥4 food groups)
	Minimum meal frequency^d^
	Consumption of iron‐rich foods
Child morbidity (last 14 days)	Symptoms of fever, cough or diarrhoea
	Cough severity^e^
	Diarrhoea severity^f^

a We created socio‐economic quintiles using a principal components analysis (PCA). Component variables were based on the Multi‐dimensional Poverty Index (Alkire & Santos 2010) and two similar principal components analyses provided there was sufficient variability in the data (Menon et al. 2000;Vyas and Kumaranayake 2006). The PCA was set to extract a single component and several iterations were run to achieve the best possible fit to the data (Field 2009). Variables in the final PCA included: household assets (fan, electricity, bicycle, motorcycle), women's literacy, fuel type (dung, wood, charcoal = most poor, gas/coal/kerosene/oil = least poor) and Land ownership (no land, <2 bighas/land mortgaged, 2–4 bighas, 4 or more bighas; one Bigha is equivalent to approximately half an acre in the study areas).

b The majority of participant households were working in the informal sector, often as daily wage labourers, and reliable income data was challenging to collect. Instead, we allocated women to three income groups (low, middle or high) based on the occupation providing the household's main source of income to reflect how lucrative these occupations would be.

c Household shocks included: a major health problem, disease epidemic, crop failure/drought/drop in production, damage to houses or crops.

d Breastfed children twice/day if 6–8 months, thrice/day if 9–23 months, non‐breastfed children four times/day.

e No cough, uncomplicated cough, cough with atypical breathing.

f No diarrhoea, uncomplicated diarrhoea, bloody diarrhoea.

We assessed candidate variables for multi‐collinearity using correlations, and by assessing tolerance and variance inflation factors. Most variables were not collinear, with the exception of father's age (collinear with mother's age); mother's age was retained given they are the main caregivers of children in the study context. In the case of variables constructed using common information (e.g. advice and treatment seeking during child illness) we retained the variable with the strongest relationship with HAZ. All remaining variables were entered simultaneously into GEE multiple linear regression models. We eliminated the least significant exposure variables (*p* > 0.1) in turn using backwards step‐wise exclusion. We included additional forward steps after each elimination to check whether previously eliminated variables had become significant (*p* ≤ 0.1) in later models.

### Ethics, consent and permissions

UK ethical approval was given for the survey by the UCL Research Ethics Committee (Application number  2163/001). At the time of survey there were no local university partners or independent research ethics committees in the study area, so local ethical approval was not possible. Informed verbal consent to participate in the survey was sought from each respondent and recorded in writing or by thumbprint.

## Results

### Participant characteristics

A total of 5184 mothers were invited to participate in the study. Of these mothers, 2619 resided in the control areas (i.e. where the data reported here originate from); 2267 (86.6%) agreed to take part.

We used data from the census conducted prior to the nutrition survey to assess non‐respondent bias. Maternal literacy was slightly higher among respondents (29.9%) compared with non‐respondents (25.3%): *χ* ^2^ (1) = 2.940, *p* = 0.086. There was no difference in the proportion of different social or ethnic groups by responder status (*χ*2 (3) = 3.251, *p* = 0.354), or in the proportion of women with a Below Poverty Line (BPL) card (65.1%): *χ* ^2^ (1) = 1.311, *p* = 0.252 (a state government issued card that enables households meeting certain poverty‐related criteria to access subsidised items such as grain).

**Figure 1:**
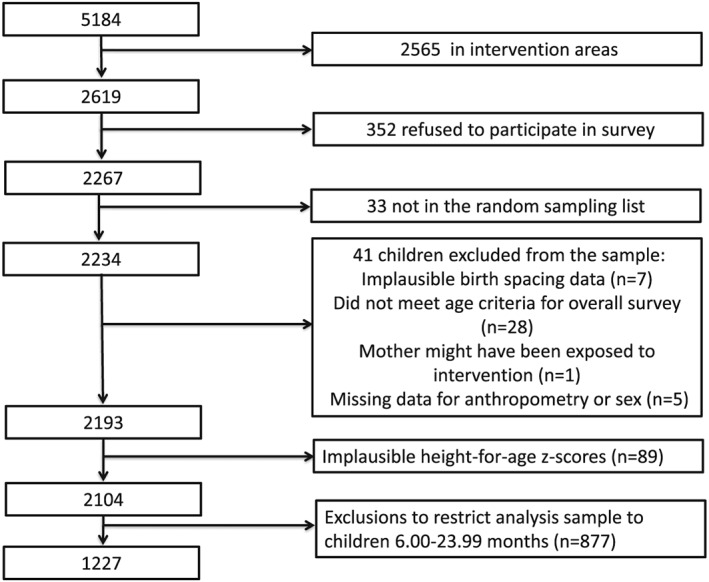
Flowchart describing the recruitment of participants and data exclusions prior to analysis.

We made additional exclusions based on eligibility criteria and data validation checks. Figure 1 describes the number of mother–child pairs available at each assessment stage, with reasons given for exclusions. The final sample available for analysis was 1227 mothers and children; Table 2 shows their characteristics.

**Table 2:** Socio‐demographic characteristics of mothers and their households (*n* = 1227)

Characteristic		% (n)
Marital status	Married	99.7 (1223)
	Co‐habiting/widowed	0.3 (4)
Age at marriage	Mean (SD)	18.4 (2.41)
	Unknown/missing % (*n*)	3.4 (42)
Status within household	Household head	0.7 (8)
	Wife	72.2 (886)
	Daughter in law	26.2 (321)
	Other relative	1.0 (12)
Maternal age (years)	Mean (SD)	26.4 (5.23)
	Unknown/missing % (*n*)	7.7 (95)
Paternal age (years)	Mean (SD)	31.1 (6.35)
	Unknown/missing % (*n*)	8.8 (108)
Religion	Sarna	44.4 (545)
	Hindu	52.9 (649)
	Christian	1.8 (22)
	Muslim	0.4 (5)
	Other	0.5 (6)
Social group	Scheduled Tribe	77.6 (952)
	Scheduled Caste	2.4 (29)
	Other Backward Class	17.4 (213)
	Other/missing	2.6 (33)
Maternal literacy	No schooling	68.4 (839)
	Primary school (first–fifth year)	3.9 (48)
	Secondary school (sixth–eighth year)	24.9 (306)
	≥Higher secondary (≥ninth year)	2.8 (34)
Land ownership^a^	No land	13.2 (163)
	<2 bighas^2^/land mortgaged	33.4 (410)
	2–4 bighas	33.9 (416)
	>4 bighas	19.3 (237)
	Missing	0.1 (1)
Cooking fuel as poverty indicator^b^	Least poor	12.1 (149)
	Most poor	87.7 (1076)
	Missing	0.2 (2)
Below poverty line card	No/Applied for	37.4 (458)
	Yes	59.9 (735)
	Missing	2.7 (34)
Income category	Lowest	82.7 (1015)
	Middle	12.9 (158)
	Highest	4.4 (54)
Socio‐economic quintile	Lowest SES group	19.5 (239)
	Second lowest SES group	11.7 (144)
	Middle SES group	21.3 (261)
	Second highest SES group	19.5 (239)
	Highest SES group	25.3 (310)
	Missing	2.7 (34)

a Bighas are a measure of land area, and vary by region: 1 bigha is about 0.5 acres in Jharkhand and Odisha.

b Wood/leaves/dung/charcoal = poorest, coal/oil/kerosene/gas = least poor Alkire & Santos, 2010).

55.7% (683/1227) of children were stunted (HAZ < −2.00). A higher proportion of boys were stunted compared with girls (57.7% and 53.7%, respectively); 30.6% (375/1227) of children were severely stunted (HAZ < −3.00): 32.7% of boys and 28.4% of girls. Stunting varied by age group: it decreased from 33% to 26.5% between 6 and 7 months but tended to increase with child age thereafter, peaking at 75.6% among children aged 22 months. A scatterplot and Pearson's correlation indicated minimal association between HAZ and weight‐for‐height *z*‐score (*r* = 0.02, *p* = 0.49).

Most women in the sample were married. Approximately three quarters were wives of the household head, and a quarter were daughters‐in‐law. More than three‐quarters (77.5%) were from indigenous groups, and nearly one‐fifth identified as Other Backward Class. Most women (68.0%) were unable to read and just over one‐fifth could read easily (21.9%). Mean maternal body mass index (BMI, weight in kg/height in m^2^) was 18.5 and ranged from 10.9 to 29.5. The BMI variable was normally distributed, but 55.7% of women were classed as underweight (BMI <18.5) (data reported in text only).

Half of households had electricity. Land ownership was variable, with 12.8% of mothers reporting that their household owned no land, and one‐fifth reporting >4 bighas (around two acres) of land. 59.5% of mothers had a BPL card, and most belonged to the lowest income category based on occupation (82.3%).

### Univariable associations

A range of basic, underlying and immediate causes of undernutrition were associated with HAZ in univariable GEE regression models. Univariable associations between all candidate predictor variables and height‐for‐age *z*‐score are presented in Additional File 1.

The variables eligible for inclusion in the multi‐variable models (backwards stepwise regression using GEE) were: socio‐economic quintile, income group, mother's and father's education, maternal BMI (included as a continuous variable), maternal height (included as a continuous variable), maternal age, district, social group, sex; self‐reported anaemia in pregnancy, women's non‐pregnancy illness or injury (previous 3 months), birth order, birth spacing, parity, season of birth, delivery location, BCG and DPT vaccinations, treatment of diarrhoea/fever/cough (last 14 days); cooking location, household overcrowding, treatment of drinking water, use of a handwashing agent, handwashing score; child minimum dietary diversity (≥4 food groups consumed in the last 24 hours), repeated bouts of diarrhoea, diarrhoea (last 14 days).

### Multivariable analysis

Results for the final model after elimination of variables with *p* > 0.10 are shown in Table 3.

**Table 3:** Final model estimates for determinants of height‐for‐age *z*‐score in children 6.00–23.99 months (*n* = 1227)

	Determinant	% (*n*) or mean (SD)	Unadjusted *β* (95%CI)	*P*‐value^a^	Adjusted *β* (95%CI)	*P*‐value^a^
Basic causes	Income group			0.007		0.065
	Lowest	82.7 (1015)	1		1	
	Middle	12.9 (158)	0.343 (0.073–0.612)	0.013	0.237 (0.033–0.441)	0.023
	Highest	4.4 (54)	0.547 (0.163–0.931)	0.005	0.253 (−0.143–0.649)	0.210
Underlying causes	Birth order			<0.001		0.001
	First born	28.0 (344)	1		1	
	Second born	23.6 (289)	0.057 (−0.155–0.269)	0.599	−0.001 (−0.243–0.240)	0.992
	Third born	17.8 (219)	−0.103 (−0.428–0.223)	0.537	−0.097 (−0.430–0.235)	0.566
	≥Fourth born	30.6 (375)	−0.446 (−0.668– −0.224)	<0.001	−0.379 (−0.651– −0.107)	0.006
	Birth spacing			0.005		0.035
	<24 months	14.8 (181)	1		1	
	≥24 months	39.8 (488)	0.464 (0.101–0.826)	0.012	0.395 (0.086–0.705)	0.012
	First child/don't know	45.5 (558)	0.452 (0.172–0.731)	0.002	0.262 (−0.26–0.551)	0.075
	Maternal body mass index	18.45 (1.84)	0.070 (0.020–0.120)	0.006	0.088 (0.039–0.137)	<0.001
	Maternal height (cm)	149.3 (5.76)	0.058 (0.035–0.080)	<0.0001	0.057 (0.036–0.078)	<0.001
	Cooking location			<0.001		<0.001
	In the house/main living area	62.6 (768)	1		1	
	In a separate room	31.4 (385)	0.268 (0.015–0.521)	0.038	0.065 (−0.156–0.287)	0.565
	Outdoors	6.0 (74)	0.823 (0.476–1.171)	<0.001	0.663 (0.348–0.977)	<0.001
	Season of birth			0.026		0.072
	Winter	20.9 (257)	1		1	
	Summer	37.2 (457)	0.043 (−0.206–0.292)	0.733	0.047 (−0.219–0.314)	0.727
	Rainy	41.8 (513)	0.285 (0.035–0.535)	0.025	0.281 (−0.006–0.568)	0.055
	Hand washing agent					
	None	80.2 (984)	1		1	
	Ash/mud/soap	19.8 (243)	0.438 (0.197–0.678)	<0.001	0.317 (0.106–0.528)	0.003
	Repeated diarrhoea					
	No	70.2 (861)	1		1	0.003
	Yes	28.4 (348)	−0.343 (−0.551– −0.135)	0.001	−0.233 (−0.387– −0.079)	
Immediate causes	Minimum dietary diversity					
	No	94.6 (1161)	1		1	
	Yes	5.4 (66)	0.496 (0.126–0.865)	0.009	0.333 (−0.065–0.732)	0.101
Other fixed demographics	Sex of child					
	Male	50.0 (614)	1		1	
	Female	50.0 (613)	0.225 (0.063–0.386)	0.006	0.271 (0.133–0.408)	<0.001

a *P*‐values are shown for the predictor variable overall, and for each category of the variable relative to the baseline category.

Income was the only basic determinant of HAZ remaining in the final adjusted model. Children from the middle income group had HAZ scores 0.237 SD units higher than those in the lower group (95% Confidence Interval/CI 0.033–0.441, *p* = 0.023). Sex remained significant: girls had higher HAZ scores than boys (*β* = 0.271, 95%CI 0.133–0.408, *p* < 0.001).

Birth order remained a strong underlying determinant of HAZ: children born fourth in the family or later had HAZ‐scores 0.379 units lower than first‐born children (95%CI −0.651– −0.107; *p* = 0.006). Several underlying predictors related to care of mothers were positively associated with child HAZ, including birth spacing ≥24 months (*β* = 0.395 95%CI 0.086–0.705, *p* = 0.012). A one unit change in maternal BMI was associated with a small increase in HAZ‐score (*β* = 0.088, 95%CI 0.039–0.137, *p* < 0.001), as was maternal height (*β* = 0.057, 95%CI 0.036–0.078, *p* < 0.001).

Cooking outdoors as opposed to in the main living area was associated with 0.663 increase in HAZ (95%CI 0.348–0.977, *p* < 0.001). Use of a handwashing agent (soap/ash/mud) compared with water alone was strongly and positively related to HAZ (*β* = 0.317, 95%CI 0.106–0.528, *p* = 0.001). Being born in the rainy season as opposed to winter had a modest positive association (*β* = 0.281, 95%CI −0.006–0.568, *p* = 0.055). Repeated diarrhoea was strongly and negatively associated with HAZ (*β* = −0.233, 95%CI −0.387– −0.079, *p* = 0.003). The single remaining immediate dietary determinant of HAZ was minimum dietary diversity (*β* = 0.333, 95%CI −0.065–0.732; *p* = 0.010).

### Missing data and multiple imputation

A small proportion of the sample had missing data for variables entered into the first backward stepwise model. Maternal age had the most missing (7.7%, *n* = 95), followed by socio‐economic quintile (2.8%, *n* = 34). The remaining variables with missing data collectively accounted for ≤3.2% of the dataset (*n* = 39).

We used multiple imputation to impute socioeconomic quintile and maternal age (constrained to be 13–55 years as per the original data range) and created 20 imputed datasets in line with published guidance (Sterne et al. 2009).

We re‐ran the backwards step‐wise process using the pooled estimates and *p*‐values from the imputed datasets. We obtained the same final model as reported in Table 3 and estimates were unchanged.

### The effect of siblings

Seventeen families (1.4% of the sample) had multiple children participating in the study. Although our model did not account for clustering at the family level, we repeated the analysis with all children, and then with one sibling randomly removed. There was no difference in the results.

### Interactions

We explored reasons for sex differences in HAZ, focusing on dietary diversity and repeated diarrhoea.

The interaction between sex and dietary diversity was significant (*p* < 0.001). Girls who did not meet dietary diversity criteria had significantly higher HAZ‐scores than male counterparts with equally poor diets (*β* = 0.190,  *p* = 0.018). We also observed a lack of association in the sub‐group of boys consuming adequately diverse diets and HAZ.

The interaction between sex and repeated diarrhoeal episodes was significant (*p* < 0.001). Girls who did not experience repeated diarrhoea had significantly higher HAZ‐scores than male counterparts (*β* = 0.202, *p* = 0.028). Boys with repeated diarrhoea had significantly worse HAZ scores than boys who did not experience repeated diarrhoea (*β* = −0.446, *p* = 0.002), but this difference was not observed in girls.

## Discussion

Our study provides the most recent data on stunting determinants among Scheduled Tribe communities in rural eastern India. We adopted a holistic approach to understanding determinants by classifying them according to the UNICEF conceptual framework. The data indicate high levels of stunting with a range of potential drivers. The strongest protective factors were cooking outdoors, adequate birth spacing (≥24 months) and handwashing with a cleansing agent. The strongest risk factors were later birth order and repeated diarrhoeal infection.

**Figure 2:**
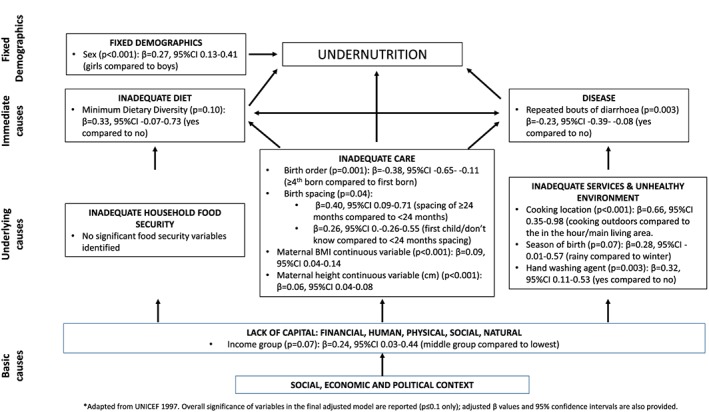
The significance of final model variables and their associations with height‐for‐age *z*‐score, mapped onto the UNICEF conceptual framework.

We discuss results for each level of the UNICEF conceptual framework below; results are also presented pictorially in Fig. 2.

### Demographic and basic determinants: higher income groups and girls were more resilient against stunting

The interaction effects between sex and HAZ suggested that girls' linear growth is less affected by inadequately diverse diets and repeated diarrhoea than boys', and that adequate dietary diversity was only significantly protective for girls. Other literature from India has suggested that girls suffer disproportionately from undernutrition because of preferential treatment of male children (Borooah, 2004), but differential feeding was not evident in our sample. It is important to reiterate that our study included predominantly indigenous participants, where females from some communities may have a higher social status than women from other social groups in India (Mitra, 2008).

Income was the only basic determinant to be retained in the final model. In this study, income may have been a proxy for disposable income to buy food and other essentials. Undernutrition is often associated with low income and a cross‐sectional survey of children under three in Andhra Pradesh also identified low wealth as a risk factor for undernutrition (Meshram et al. 2011). Another study from Eastern India observed a weaker income gradient in undernutrition, with only children from the richest wealth group deriving significant benefits. The authors noted that small increases in income may not lead to substantial reductions in undernutrition without additional gains in health and education (Bhagowalia et al. 2012). The lack of significant associations between stunting, having no education or belonging to lower socio‐economic quintiles was surprising. National‐level analyses by Corsi et al. (2015) found that having no education and belonging to the poorest wealth quintile were two of the strongest risk factors for stunting, and these associations have long been documented in ecological studies (Black et al., 2013). We included many more variables related to underlying determinants of stunting compared with Corsi et al.'s (2015) analysis. It is possible that these ‘washed out’ the effects of some basic determinants, or that our sample was too small to detect their effects.

### Underlying ‘health environment’ determinants: handwashing, cooking outdoors and being born during the rainy season were protective against stunting

Handwashing with a cleansing agent was strongly protective against stunting. Systematic reviews have highlighted the potential of handwashing to reduce diarrhoea by 40–48% (Cairncross et al. 2010, Fewtrell et al. 2005, WHO & UNICEF, 2009). Another review suggested that handwashing also reduces the risk of viral and bacterial pneumonia (Luby et al. 2005). Intestinal worms are highly prevalent in our study areas, and there is a link between helminth infections and child undernutrition (Awasthi et al. 2008, Hall et al. 2008). Worms can be transferred through the faecal–oral route, which would be disrupted by good handwashing practices. A cross‐sectional survey in rural Andhra Pradesh identified not using soap for handwashing as one of the strongest predictors of young child stunting (Meshram et al. 2011).

Cooking outdoors rather than the main living area appeared to be strongly protective against stunting. A likely explanation is that cooking outdoors reduces exposure to harmful indoor air pollutants from the burning of biomass fuels. The use of biomass fuels for cooking was high (>87%), and most people cooked over an open fire (>85%). Cooking tasks are usually performed by women in the study areas, thereby exposing them, unborn and young children to biomass fuel smoke more than other family members (Bruce et al. 2000, Duflo et al. 2008). National data (1998–9) showed that severe stunting was 84% higher in biofuel burning households and child anaemia prevalence was higher compared with households using cleaner fuels, after adjusting for tobacco smoke, maternal education, nutrition and recent illness (Mishra and Retherford, 2007). Demographic and health surveys from seven developing countries found biofuel exposure was associated with a 0.13 lower HAZ‐score than for non‐biofuel households, after confounder adjustment (Kyu et al. 2009). One known mechanism is that indoor air pollution increases the risk of acute respiratory infections, which can lead to stunting (Bruce et al. 2000). There is also consistent epidemiological evidence that indoor air pollution can cause low birth weight (LBW) (Bruce et al. 2000). A cohort study from South India measured children from birth to 6 months at two‐week intervals and identified a 49% increased risk of LBW and a 30% higher risk of stunting at 6 months in households using wood and/or dung as their main fuel compared with cleaner fuels (Tielsch et al. 2008). Much of this LBW may be attributable to intrauterine growth restriction: exposure to particulate matter and other noxious substances in pregnancy can increase the risk or exacerbate the problem in already vulnerable populations with high levels of maternal underweight and anaemia (Tielsch et al. 2008). Although there appears to be a strong protective influence of cooking outdoors on height‐for‐age, our explanation that this is because of lowered exposure to indoor air pollution remains speculative. We do not have data on other important aspects of indoor air pollution (e.g. exposure time, use of fires/stoves inside for other reasons, tobacco use or air quality measurements) which could partially account for this finding. Paternal smoking has been linked to stunting in previous studies, although the effect seems to be weaker than for burning biofuels (Duflo et al. 2008).

Being born in the rainy season was protective against stunting compared with being born in the winter. The relative disadvantage to winter‐born children could be because of worsened intrauterine growth restriction and stunting in early life from extra exposure to biofuel smoke to keep warm in the winter (Bruce et al. 2000). There are also seasonal peaks in respiratory infections in winter that could contribute to stunting (Luby et al. 2005).

### Underlying maternal care determinants: taller mothers, mothers with higher BMIs and those who spaced births by ≥24 months had children with significantly higher height‐for‐age

Maternal BMI and height were modestly and positively related to HAZ. More than half of women (55.7%) were underweight in this sample (BMI <18.5), while 44.1% were in the healthy BMI range (BMI 18.5–24.9), and 0.2% were overweight (BMI 25.0–29.9); none of the women were obese (BMI ≥30). Low maternal BMI is a known risk factor for intrauterine growth restriction and subsequent growth faltering (Black et al. 2013b). Maternal underweight can also affect child growth through reduced micronutrient content of breast milk, in particular vitamin A, which is important because infants have low stores at birth (Black et al. 2013b, Black et al. 2013a). Low maternal BMI may also reflect poor dietary intake and adequacy, and low food availability, which could partly explain this association. Corsi et al.'s analysis of NFHS‐3 data also found strong associations between stunting and low maternal stature (<145 cm) (OR: 4.2, 95% CI: 3.5–5.0) as well as maternal BMI (OR: 1.4, 95% CI: 1.2–1.6).

Adequate birth spacing (≥24 months) appeared strongly beneficial for HAZ. This corresponds with NFHS‐2 and NFHS‐3 analyses that identified birth spacing <24 months as a risk factor for stunting (Som et al. 2007, Debnath and Bhattacharjee, 2014). In a recent analysis of NFHS‐3 data focusing on Scheduled Tribe children, those whose mothers had short pregnancy intervals (<2 years) had a twofold increase in the odds of stunting (adjusted OR 2.0, 95%CI 11.2–3.6) (Debnath and Bhattacharjee, 2014). Mechanisms include compromised nutrition for the first‐born child through early interruption of breastfeeding, and, for the second child, a greater risk of LBW (Dewey and Cohen, 2007, Wendt et al. 2012). One review found that the association between birth spacing and child growth was inconsistent: about half of the studies found a positive association where intervals of ≥36 months equated to reduced stunting risks of 10–50% (Dewey and Cohen, 2007). The risks associated with maternal undernutrition and anaemia were also mixed, and not all studies adjusted for obvious confounders, such as breastfeeding. We were unable to collect objective measures of anaemia during pregnancy and relied on self‐report, which was univariably associated with HAZ despite probably being an underestimate. In the NFHS‐3 survey prevalence of any anaemia was 61.2% in Orissa, and 69.5% in Jharkhand (Debnath and Bhattacharjee, 2014). Dewey and Cohen (2007) consider the ‘recuperative’ interval (when women are neither pregnant or breastfeeding) as potentially more relevant to maternal health than pregnancy or birth intervals (Dewey and Cohen, 2007). A more recent meta‐analysis relating inter‐pregnancy interval to birth outcomes found that intervals of <12 months significantly increased the risks of prematurity, LBW, stillbirths and early neonatal deaths (Wendt et al. 2012). Inadequate birth spacing could be a sign of unmet need for family planning, and this highly nutrition‐sensitive aspect of health provision should be a priority in underserved areas.

### Underlying child care determinants: later birth order is a significant risk factor for stunting

Children born later in large families were at greater risk of stunting in this sample, particularly children born ≥fourth compared with first‐borns. This is consistent with NFHS data: in the NFHS‐1, ≥third born children had a 1.26–1.56 greater risk of severe stunting (Mishra and Retherford, 2007) and in the NFHS‐3, there was an elevated risk for ≥sixth born compared with first‐born children from Scheduled Tribes (Debnath and Bhattacharjee, 2014). Later birth order (and greater parity) is likely to stretch household resources and undermine the effectiveness of caring practices. In addition to addressing unmet need for family planning in the study areas, interventions to counteract the negative effects of later birth order could address sibling‐to‐sibling care. Not only does this increase the likelihood of infections and sub‐optimal feeding, as children may be less likely to understand these issues than adults, but child care responsibilities are a common reason for female siblings leaving education prematurely (Sengupta and Jaba, 2002). There has been a recent attempt to increase the availability of crèches to counteract this problem, and they may be incorporated into Integrated Child Development Service reforms (Indian Planning Commission, 2011).

### Underlying child health status: repeated diarrhoeal infection was associated with lower height‐for‐age

Repeated diarrhoea infection was negatively related to HAZ, consistent with previous studies. A multi‐country longitudinal study identified a dose–response relationship between each day of diarrhoea in the first two years of life and stunting at 24 months, accounting for 18% of stunting (Checkley et al. 2008). A Brazilian cohort study from birth to 24 months also found the duration of diarrhoeal episodes was important: 7–13 days significantly worsened HAZ‐scores relative to acute episodes (<7 days) and prolonged episodes doubled the risk of developing persistent diarrhoea (≥14 days) in later childhood (Moore et al. 2010). Each day of diarrhoea amounted to a day of missed opportunity for linear growth, and if prolonged, minimised the possibility for catch‐up growth.

Diarrhoea may have indirect effects on child growth in areas with pre‐existing high mortality and prevalent undernutrition. One study attributed 26% of acute lower respiratory infections to recent diarrhoea in a Ghanaian cohort with high baseline levels of undernutrition and mortality, but they did not observe this effect in a better‐nourished Brazilian cohort with low mortality. In other words, for particularly vulnerable populations there may be an additional pathway to undernutrition and death from diarrhoea via elevated respiratory infection risk (Schmidt et al. 2009). The authors suggest that the mechanism could operate through acute micronutrient loss because of diarrhoea and subsequent immune system impairment, dehydration and immobilisation that creates a window for opportunistic infections. Extra efforts dedicated to diarrhoea reduction could thus also reduce incidence of acute respiratory infections in malnourished populations.

### Immediate determinants: dietary diversity was protective against stunting in girls but not boys

Minimum child dietary diversity was protective against stunting for girls, but not boys. Only 5.4% of children had adequately diverse diets (≥4 food groups the previous day) which is lower than the 15.2% reported in the NFHS‐3 (Schmidt et al. 2009). Micronutrients are essential for growth and development in the first two years of life. From 6 months the majority of iron, zinc and Vitamin B6 are required from food, even with continued breastfeeding, and the proportion of energy, protein and essential fatty acids also increases. Animal source foods are important because they are rich in protein and micronutrients; a lack of these foods is a risk factor for stunting and iron‐deficiency anaemia. The very low dietary diversity and iron‐intake among indigenous children is worrying and probably reflects the late introduction of complementary foods as well as poor diet among those who have been introduced to those foods. The low consumption of iron‐rich foods in our sample may be because of prohibitive cost or cultural inappropriateness. Recent NFHS‐3 analyses found minimum dietary diversity was the infant and young child feeding indicator that was most strongly associated with stunting and underweight (*p* < 0.05) (Menon et al. 2015), and was also in the top five risk factors for stunting (Corsi et al. 2015).

Late introduction of complementary foods probably contributed to the low percentage of children 6–24 months consuming an adequately diverse diet. There are many reasons for late introduction, including seasonal factors relating to food availability, postponing the celebration associated with children taking their first food (because of the high costs of hosting), caregivers being unaware their child has reached six months, and increasing food prices (Meshram et al. 2011, Dewey and Brown, 2003). One NFHS‐3 analysis identified stunting as a determinant of late introduction of complementary foods, and suggested that mothers of stunted children may not have felt their child was ‘ready’ for food because they were small (Patel et al. 2012).

### Limitations

Key limitations of our study include lack of variability or data for other potential stunting determinants, the cross‐sectional design, potential residual confounding and limited generalizability to other indigenous communities in India.

There was insufficient variability in many water, sanitation and hygiene measures, and these could not be included in the analyses. For example, most respondents practiced unsafe disposal of children's faeces (97.3% throw faeces outside), just 0.9% reported handwashing before preparing food and 99.2% of caregivers reported open defecation. These are probably important determinants of undernutrition but could not be fully examined. One study from Peru (Checkley et al. 2004) and a multi‐country analysis of DHS data from eight low‐income countries (Esrey, 1996) have highlighted that improved sanitation has a greater impact on children's linear growth than improved drinking water and safe water storage alone. The authors of the Peruvian study found that the health benefits of improved water supplies were not fully realised unless safe water storage practices and improved sanitation were in place. The DHS analysis indicated that improved water supplies did not significantly increase child height unless improved sanitation was available, but also identified a synergistic effect between improved water and sanitation on child height that was greater than if only one of the services was present. It is likely that improved coverage of sanitation in the study areas would strengthen the effectiveness of water treatment and hand washing as stunting reduction interventions.

We did not measure water storage, which is an important dimension of drinking water safety; it will be useful to consider this in future work. A common causal factor in undernutrition is environmental enteropathy, for which only invasive biomarker measures are currently available. This would not have been appropriate for our survey, but other researchers are developing urinary, faecal and blood‐based markers which may be more acceptable (Prendergast and Kelly, 2012).

The question asking whether children suffered from repeated attacks of diarrhoea did not quantify the duration or severity of episodes, and only captured mothers' perceptions of whether the problem was recurring. In the case of recurrent diarrhoea, longitudinal studies have shown that the duration of previous diarrhoeal episodes is important, and that prolonged episodes have more serious implications for linear growth than shorter, acute episodes (Moore et al. 2010). It was not possible to collect detailed information about recurrent diarrhoea episodes in this cross‐sectional survey.

The cross‐sectional design of our study means that we are unable to infer causal associations between the determinants and HAZ. There may be causality in both directions for particular variables: for example, positive feedback between undernutrition and morbidities such as diarrhoea would be expected. Some of the associations found in our analyses may also be accounted for or modified by unmeasured confounding.

Finally, our sample was drawn from purposively selected clusters in two states of eastern India, which limits the generalizability of our findings. However a recent NFHS‐3 analysis focusing on stunting determinants among of 1606 Scheduled Tribe children 6–23 months identified stunting determinants that are aligned with those we found, including child age, maternal stunting and short pregnancy intervals (<2 years) (Debnath and Bhattacharjee, 2014).

## Source of funding

The research was supported by the Big Lottery Fund, grant reference number: IS/2/010281409, and by a UK Medical Research Council doctoral fellowship to JC Saxton. The funder had no role in designing the study, data collection and analysis, the decision to publish or the preparation of this manuscript.

## Conflicts of interest

The authors declare that they have no conflicts of interest.

## Contributor statement

JS, AP, NN and PT designed the study. All authors contributed to the design of the questionnaire. JS, SR, RM and RG carried out the anthropometry and questionnaire training, and supervised data collection. JS carried out the analyses and wrote the first draft of the article, with help from AP. All authors contributed to subsequent manuscript revisions.

## Supporting information

Supporting info item
