# Supplementary material for: Handwashing, sanitation and family planning practices are the strongest underlying determinants of child stunting in rural indigenous communities of Jharkhand and Odisha, Eastern India: a cross‐sectional study
Source: Matern Child Nutr. 2016 Jun 27;12(4):869–84. doi: 10.1111/mcn.12323 (PMC5053246; doi:10.1111/mcn.12323)
Supplement: Supplementary file 1 — Supporting info item [file MCN-12-869-s001.docx]

| Additional File 1: Univariable associations between candidate predictor variables and Height-for-age Z-score | | |
| --- | --- | --- |
| Predictor | Unadjusted β (95%CI) | P |
| **AGE AND SEX VARIABLES** |  |  |
| Child sex 0=boy, 1=girl | 0.225 (0.063-0.386) | 0.006 |
| Maternal age (years) | -0.033 (-0.051—0.015) | <0.001 |
| Paternal age (years) | -0.024 (-0.040—0.007) | 0.005 |
| **BASIC CAUSES OF UNDERNUTRITION** | | |
| Socioeconomic quintile  0-lowest, 1=second lowest  0-lowest, 1=middle  0-lowest, 1=second highest  0-lowest, 1=highest | Wald=12.106  0.205 (-0.076-0.486)  0.267 (-0.137-0.670)  0.587 (0.109-1.065)  0.578 (0.195-0.961) | 0.017  0.153  0.195  0.016  0.003 |
| Income group  0=poorest, 1=middle  0=poorest, 1=richest | Wald=9.859  0.343 (0.073-0.612)  0.547 (0.163-0.931) | 0.007  0.013  0.005 |
| Mother’s education  0=no schooling, 1=primary school  0=no schooling, 1= secondary school  0=no schooling, 1= ≥higher secondary | Wald=19.899  0.131 (-0.114-0.376)  0.390 (0.168-0.612)  0.902 (0.392-1.412) | <0.001  0.296  0.001  0.001 |
| Father’s education  0=no schooling, 1=primary school  0=no schooling, 1= secondary school  0=no schooling, 1= ≥higher secondary | Wald=17.119  0.047 (-0.216-0.310)  0.253 (-0.037-0.542)  0.428 (0.223-0.634) | 0.001  0.728  0.087  <0.001 |
| District  0=West Singhbhum, 1=Saraikela  0=West Singhbhum, 1=Keonjhar | Wald=5.397  0.314 (0.011-0.617)  -0.098 (-0.502-0.307) | 0.069  0.043  0.636 |
| Religion  0=Sarna, 1=Hindu  0=Sarna, 1= Christian/Muslim/other | Wald=0.453  0.040 (-0.271-0.350)  -0.185 (-0.855-0.484) | 0.797  0.803  0.588 |
| Social group  0=Scheduled Tribe, 1=Scheduled Caste  0=Scheduled Tribe, 1=Other Backward Class  0=Scheduled Tribe, 1=Other | Wald=7.742  0.373 (-0.055-0.801)  0.354 (0.078-0.630)  0.106 (-0.661-0.873) | 0.052  0.087  0.012  0.787 |
| **UNDERLYING CAUSES OF UNDERNUTRITION** | | |
| ***Food security (household shocks in the previous 12 months)*** | | |
| Disease epidemic 0=no, 1=yes | 0.013 (-0.190-0.216) | 0.900 |
| Major household health problem 0=no, 1=yes | -0.106 (-0.523-0.312) | 0.620 |
| Crop failure 0=no, 1=yes | 0.069 (-0.231-0.368) | 0.653 |
| Damage to houses or crops by elephants 0=no, 1=yes | -0.051 (-0.414-0.312) | 0.782 |
| Any household shock 0=no, 1=yes | -0.050 (-0.324-0.224) | 0.720 |
| ***Care for children*** |  |  |
| Early initiation of breastfeeding: 0=no, 1=yes | 0.114 (-0.170-0.398) | 0.431 |
| Bottle feeding 0=no, 1=yes | 0.262 (-0.080-0.603) | 0.133 |
| Pre-lacteal feeds 0=no, 1=yes | 0.157 (-0.326-0.640) | 0.523 |
| Colostrum discarding 0=no, 1=yes | 0.138 (-0.180-0.455) | 0.396 |
| BCG immunisation 0=no, 1=yes | 0.353 (-0.025-0.731) | 0.067 |
| DPT immunisations (3) 0=no, 1=yes | 0.299 (0.021-0.578) | 0.035 |
| Polio immunisations (3) 0=no, 1=yes | 0.242 (-0.106-0.590) | 0.173 |
| Feeding frequency during diarrhoea, fever, cough  0=none/less, 1=same/more  0-none/less, 2=n/a (no diarrhoea, fever , cough) | Wald=2.490  0.187 (-0.061-0.435)  0.225 (-0.063-0.512) | 0.288  0.140  0.129 |
| Liquids given during diarrhoea, fever, cough  0=none/less, 1=same/more  0-none/less, 2=n/a (no diarrhoea, fever, cough) | Wald=5.377  -0.091 (-0.584-0.402)  0.148 (-0.199-0.495) | 0.068  0.717  0.403 |
| Treatment seeking for diarrhoea, fever, cough  0=no, 1=yes  0=no, 1=n/a (no diarrhoea, fever or cough) | Wald=20.257  0.522 (0.266-0.779)  0.486 (0.256-0.715) | <0.001  <0.001  <0.001 |
| ORS given for diarrhoea (last 14 days)  0=no, 1=yes  0=no, 1=n/a no diarrhoea | Wald=5.697  -0.162 (-0.643-0.318)  0.141 (-0.096-0.378) | 0.058  0.508  0.245 |
| Birth order  First born, 1=Second born  0=First born, 1=Third born  0=First born, 1=≥Fourth born | Wald=21.287  0.057 (-0.155-0.269)  -0.103 (-0.428-0.223)  -0.446 (-0.668- -0.224) | <0.001  0.599  0.537  <0.001 |
| ***Care for mothers*** | | |
| Number of children born  0=one, 1=two-three children  0=one, 1=≥4 children | Wald=6.751  0.067 (-0.280-0.413)  -0.296 (-0.562- -0.030) | 0.034  0.706  0.029 |
| Birth spacing  0=<24 months, 1=≥24 months  0=<24 months, 1-Don’t know/missing | Wald=10.619  0.464 (0.101-0.826)  0.452 (0.172-0.731) | 0.005  0.012  0.002 |
| Self-reported anaemia in pregnancy (0=no, 1=yes) | -0.280 (-0.539- -0.022) | 0.033 |
| Self-reported malaria in pregnancy (0=no, 1=yes) | -0.244 (-0.587-0.098) | 0.162 |
| Iron tablets in pregnancy (0=no, 1=yes) | -0.006 (-0.258-0.247) | 0.966 |
| Quantity of iron tablets in pregnancy  0=no tablets, 1=<50 tablets  0=no tablets, 1=≥50 tablets | Wald=4.291  -0.133 (-0.384-0.118)  0.085 (-0.216-0.385) | 0.117  0.299  0.581 |
| Food consumption in pregnancy  (0=less, 1=same/more than usual) | 0.001 (-0.284-0.286) | 0.995 |
| Maternal Body Mass Index | 0.070 (0.020-0.120) | 0.006 |
| Maternal height (cm) | 0.058 (0.035-0.080) | <0.001 |
| Physical illness/injury in the last three months  (non-pregnancy) 0=no, 1=yes | -0.229 (-0.452- -0.005) | 0.045 |
| Psychological distress  (K10 scores >15: 0=no, 1=yes) | 0.153 (-0.112-0.418) | 0.259 |
| ***Underlying child health issues*** | | |
| Repeated diarrhoea | -0.343 (-0.551- -0.135) | 0.001 |
| Repeated fever | -0.193 (-0.428-0.042) | 0.108 |
| Repeated cough | -0.104 (-0.319-0.112) | 0.344 |
| ***Health environment and services*** | | |
| Place of delivery  0=Home/providers home/other, 1=govt facility  0=Home/providers home/other, 1=private facility | Wald=14.032  0.228 (0.053-0.403)  0.590 (0.246-0.935) | 0.001  0.011  0.001 |
| Antenatal visit (0=no, 1=yes) | 0.181 (-0.097-0.458) | 0.202 |
| Postnatal visit (0=no, 1=yes) | 0.129 (-0.075-0.3330 | 0.216 |
| Growth monitoring  (0=less than once/month, 1=≥once/month | 0.050 (-0.188-0.289) | 0.680 |
| Food rations received via AWW  0=rarely or never, 1=daily or weekly  0=rarely or never, 1=monthly | Wald=0.932  0.090 (-0.261-0.441  0.143 (-0.182-0.468) | 0.627  0.614  0.387 |
| Living area:  0= >3 per sleeping room, 1=≤3 per sleeping room | 0.244 (0.049-0.438) | 0.014 |
| Cooking location  0=in the house, 1=in a separate room  0=in the house, 1=outdoors | Wald=21.596  0.268 (0.015-0.521)  0.823 (0.476-1.171) | <0.001  0.038  <0.001 |
| Season of birth  Season of birth_1: 0=winter, 1=summer  Season of birth_2: 0=winter, 1=rainy | Wald=7.324  0.043 (-0.206-0.292)  0.285 (0.035-0.535) | 0.026  0.733  0.025 |
| Source of drinking water:  0=unimproved, 1=improved | 0.153 (-0.089-0.396) | 0.215 |
| Treatment of drinking water  0=none, 1=physical or chemical | 0.255 (0.008-0.502) | 0.043 |
| Time taken to collect drinking water  0=>30, 1=≤30 minutes | 0.047 (-0.147-0.241) | 0.633 |
| Disposal of children’s faeces  0=unsafe practices only, 1=some safe practices | -0.174 (-0.550-0.202) | 0.364 |
| Hand washing agent: 0=none, 1=ash/mud/soap | 0.438 (0.197-0.678) | <0.001 |
| Hand washing occasions using soap (0=no, 1=yes)  Before preparing food  Before feeding a child  After defecation  After cleaning a child who has defecated  Before eating  Hand washing score based on the above (0-5) | -0.036 (-1.574-1.503)  -0.719 (-0.898- -0.540)  0.518 (0.275-0.760)  0.509 (0.106-0.912)  0.029 (-0.299-0.357)  0.237 (-0.007-0.480) | 0.964  <0.001  <0.001  0.013  0.862  0.057 |
| **IMMEDIATE CAUSES OF UNDERNUTRITION** | |  |
| ***Dietary intake/breastfeeding (previous 24 hours)*** | | |
| Predominant breastfeeding (0=no, 1=yes) | 0.040 (-0.229-0.308) | 0.772 |
| Minimum dietary diversity (0=no, 1=yes) | 0.496 (0.126-0.865) | 0.009 |
| Minimum meal frequency (0=no, 1=yes) | -0.092 (-0.277-0.092) | 0.327 |
| Consumption of iron-rich foods (0=no, 1=yes) | 0.098 (-0.228-0.424) | 0.556 |
| Age-appropriate breastfeeding (0=no, 1=yes) | -0.074 (-0.316-0.168) | 0.547 |
| ***Health status*** |  |  |
| Diarrhoea (last 14 days) 0=no, 1=yes | -0.203 (-0.383- -0.023) | 0.027 |
| Fever (last 14 days) 0=no, 1=yes | -0.092 (-0.302-0.728) | 0.394 |
| Cough (last 14 days) 0=no, 1=yes | -0.080 (-0.306-0.146) | 0.489 |
| Diarrhoeal severity (last 14 days)  0=no diarrhoea, 1=diarrhoea, no blood  0=no diarrhoea, 1=diarrhoea, blood present | Wald=4.888  -0.203 (-0.401- -0.005)  -0.204 (-0.715-0.306) | 0.087  0.045  0.433 |
| Cough severity (last 14 days)  0=no, 1=yes  0-no, 1=yes + abnormal breathing | Wald=1.294  -0.001 (-0.324-0.322)  -0.115 (-0.341-0.110) | 0.524  0.995  0.891 |
